# Biopsychosocial Profiles of Patients With Cardiac Disease in Remote Rehabilitation Processes: Mixed Methods Grounded Theory Approach

**DOI:** 10.2196/16864

**Published:** 2021-11-03

**Authors:** Marjo-Riitta Anttila, Anne Soderlund, Teemu Paajanen, Heikki Kivistö, Katja Kokko, Tuulikki Sjögren

**Affiliations:** 1 Faculty of Sport and Health Sciences University of Jyväskylä Jyväskylä Finland; 2 Department of Physiotherapy University of Mälardalen Västerås Sweden; 3 Finnish Institute for Health and Welfare Helsinki Finland

**Keywords:** coronary disease, experience, biopsychosocial model, digital cardiac rehabilitation, mixed methods grounded theory, web-based program, physical activity, self-efficacy, quality of life

## Abstract

**Background:**

Digital development has caused rehabilitation services and rehabilitees to become increasingly interested in using technology as a part of rehabilitation. This study was based on a previously published study that categorized 4 groups of patients with cardiac disease based on different experiences and attitudes toward technology (e-usage groups): *feeling outsider*, *being uninterested*, *reflecting benefit*, and *enthusiastic using.*

**Objective:**

This study identifies differences in the biopsychosocial profiles of patients with cardiac disease in e-usage groups and deepen the understanding of these profiles in cardiac rehabilitation.

**Methods:**

Focus group interviews and measurements were conducted with 39 patients with coronary heart disease, and the mean age was 54.8 (SD 9.4, range 34-77) years. Quantitative data were gathered during a 12-month rehabilitation period. First, we used analysis of variance and Tukey honestly significant difference test, a *t* test, or nonparametric tests—Mann–Whitney and Kruskal–Wallis tests—to compare the 4 e-usage groups—*feeling outsider*, *being uninterested*, *reflecting benefit*, and *enthusiastic using*—in biopsychosocial variables. Second, we compared the results of the 4 e-groups in terms of recommended and reference values. This analysis contained 13 variables related to biomedical, psychological, and social functioning. Finally, we formed biopsychosocial profiles based on the integration of the findings by constant comparative analysis phases through classic grounded theory.

**Results:**

The biomedical variables were larger for waistline (mean difference [MD] 14.2; 95% CI 1.0-27.5; *P*=.03) and lower for physical fitness (MD −0.72; 95% CI −1.4 to −0.06; *P*=.03) in the *being uninterested* group than in the *enthusiastic using* group. The *feeling outsider* group had lower physical fitness (MD −55.8; 95% CI −110.7 to −0.92; *P*=.047) than the *enthusiastic using* group. For psychosocial variables, such as the degree of self-determination in exercise (MD −7.3; 95% CI −13.5 to −1.1; *P*=.02), the *being uninterested* group had lower values than the *enthusiastic using* group. Social variables such as performing guided tasks in the program (*P*=.03) and communicating via messages (*P*=.03) were lower in the *feeling outsider* group than in the *enthusiastic using* group. The *feeling outsider* and *being uninterested* groups had high-risk lifestyle behaviors, and adherence to the web-based program was low. In contrast, members of the *being uninterested* group were interested in tracking their physical activity. The *reflecting benefit* and *enthusiastic using* groups had low-risk lifestyle behavior and good adherence to web-based interventions; however, the *enthusiastic using* group had low self-efficacy in exercise. These profiles showed how individuals reflected their lifestyle risk factors differently. We renamed the 4 groups as *building self-awareness*, *increasing engagement*, *maintaining a healthy lifestyle balance,* and *strengthening self-confidence.*

**Conclusions:**

The results facilitate more effective and meaningful personalization guidance and inform the remote rehabilitation. Professionals can tailor individual web-based lifestyle risk interventions using these biopsychosocial profiles.

## Introduction

### Background

Coronary heart disease (CHD) affects working-age populations and is the most common cause of death globally [1,2]. The main risk factors for CHD include age-related, gender-related, lifestyle-related, and socially-related risk factors [3-6]. Biomedical risk factors include smoking, high blood pressure and high cholesterol, obesity, type 2 diabetes, inappropriate diet, and sedentary behaviors [3-5]. Psychosocial factors, such as depression, lack of social support, stress, and personality type, have also been shown to affect the management of cardiovascular risks [7,8]. Cardiac rehabilitation focuses on decreasing patients’ biomedical and lifestyle risk factors and increasing psychosocial management, physical activity counseling, and exercise training [3,4,9-11]. Currently, technology can provide an opportunity for individually tailored rehabilitation, irrespective of time and place [12]. Digital development has led patients with cardiac disease to become increasingly interested in using technology [13]. Therefore, theory- and evidence-based behavior change methods [13,14] and approaches have been gradually developed in web-based programs for cardiac rehabilitation [15-19].

It is a widely held view that most people find it difficult to change their health behaviors [20]. Therefore, it is important to understand how physical, psychological, and social factors contribute to behavioral change [21]. This study is based on behavioral medicine from a biopsychosocial model perspective [21-24] to understand the lifestyle risk management of patients with cardiac disease. Behavioral medicine integrates behavioral and biomedical knowledge on health and illness and applies this information, for example, to the counseling process of remote rehabilitation [24-26]. This study is also founded on behavior theories in behavioral medicine, that is, theories of learning (social cognitive theory [SCT] and self-efficacy) and motivation in exercise contexts (self-determination and self-regulation).

SCT focuses on the dynamic interaction of personal, environmental, and health behavior factors [27,28]. Part of the theory relates to health behavior self-efficacy, which refers to personal efficacy and guides how well people motivate themselves and their thoughts and actions [28]. Several studies have shown that low self-efficacy in health behaviors is associated with increased cardiovascular risk behavior [29,30]. On the other hand, individuals with higher self-efficacy are more effective in managing their cardiovascular risk behavior [31,32]. Moreover, high self-efficacy in using technology may increase the participation of individuals in web-based rehabilitation settings [32,33].

Self-determination theory focuses on the degree to which human motivation, development, and personality functioning occur within social contexts [34]. This theory has been used to examine behavior self-regulation [35] in cardiac rehabilitation [36,37]. Research has shown that decreases in external regulation and increases in intrinsic motivation may positively affect the physical behavior of patients with cardiac disease [36]. Self-determination theory represents a framework for understanding the exercise motivation of patients with cardiac disease.

Biopsychosocial profiles have been studied in the context of disease [38-42]; however, research has rarely looked at the biopsychosocial profiles of patients with cardiac disease in web-based rehabilitation settings. It is important to identify the biopsychosocial profiles of patients with cardiac disease to which web-based interventions can be tailored individually. The digital context offers an expanded means of understanding individual experiences with digital health solutions [22].

### Objective

The purpose of this study is to enhance the understanding of biopsychosocial behaviors for the 4 previously defined different e-usage groups [43]. In our previous qualitative study, we identified 4 different e-usage groups using the Glaser mode of the grounded theory approach. These groups were *feeling outsider*, *being uninterested*, *reflecting benefit*, and *enthusiastic using* [43]. The qualitative study shows that patients with cardiac disease were different as technology users in technology experiences and attitudes toward technology and web-based guidance. Patients who felt outsiders and were not interested in technology needed more face-to-face guidance for rehabilitation, whereas patients who reflected the benefits and were enthusiastic about using technology felt that web-based coaching is sufficient support in rehabilitation [43].

In this study, we identify biopsychosocial variables related to CHD risk factors. The main biomedical and physical risk factors for CHD include physical inactivity and obesity. Psychological risk factors, such as depression, low psychological quality of life, and poor self-efficacy and behavioral control, are associated with increased CHD and risk behavior. Social determinants such as social isolation and low participation are also well-known risk factors for CHD.

In light of the previous study [43], we hypothesize that there would be differences among the 4 e-usage groups—*feeling outsider*, *being uninterested*, *reflecting benefit*, and *enthusiastic using* [43]—in each of the biomedical, psychological, and social areas. Propositions for differences among the 4 groups are as follows:

Proposition 1: The *feeling outsider* group might benefit from developing self-efficacy in physical activity and adequate positive support, as individuals in this group consider themselves as outsiders and find technology fearsome.

Proposition 2: The b*eing uninterested* group might benefit from weight management and physical activity self-monitoring with reminders and prompts, as they feel externally motivated.

Proposition 3: The *reflective benefit* groups might benefit from easy-to-use and interactive technology, as their interest is maintained by technology with personalized information and interactive tracking tools.

Proposition 4: The *enthusiastic users* group might benefit from empowering their self-efficacy and personalized lifestyle feedback, as they have a positive technology mastery experience.

## Methods

### Study Approach

We used a mixed methods grounded theory (GT) approach in this study. During the previous study in our research project, we used the Glaser inductive GT approach and open coding strategies [44]. We derived the contents of patients’ experiences with modern technology from survey responses and focus group interviews [43]. Methodologically, this study aims to further understand our previous qualitative results on the 4 e-usage groups [43] and to deepen the analysis to the core category level. Therefore, we decided to apply a qualitative and quantitative combination of the GT approach [45]. The GT methodology with quantitative data has been used across disciplines [46-49] and in health sciences because of the diversity of study questions [50]. However, it has not been used in rehabilitation settings for patients with CHD. Mixed data, methods, and techniques facilitated a balanced theory generation [49]. This helped us identify a biopsychosocial profile within 4 e-usage groups—*feeling outsider*, *being uninterested*, *reflecting benefit*, and *enthusiastic using*—and generate substantive theory.

### Study Design

This study is part of a larger project, with a cluster randomized controlled trial of a rehabilitation intervention registered in the ISRCTN registry (ISRCTN61225589). The ethics committee of the Central Finland Health Care District approved the study. The intervention assessing the effect of additional remote technology rehabilitation on patients with CHD was conducted from 2015 to 2017 in a rehabilitation center in the middle of Finland, where the Social Insurance Institution of Finland arranges regular cardiac rehabilitation courses. Before 12 months of rehabilitation, the participants were randomly allocated into intervention groups (n=10 in each group) with scheduled rehabilitation sessions for each group. Groups were randomized in pairs into the experimental groups (n=4 groups, which included 1 pilot group of experiments) and control groups (n=3 groups).

**Figure 1:**
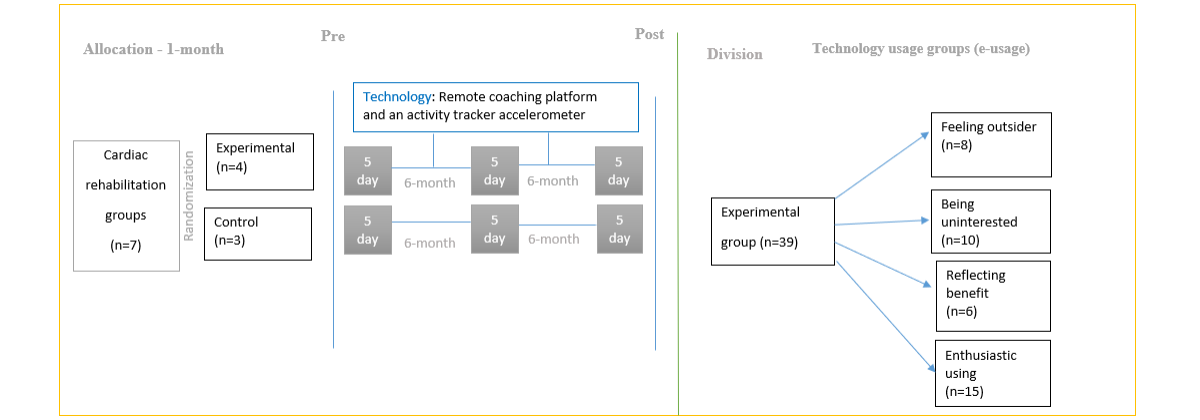
The study design of the 12-month cardiac rehabilitation (15-day) intervention within used digital health tools and division into 4 technology use groups (e-usage).

In this study, participants were from the 4 experimental groups that used digital health tools in addition to the traditional 12-month cardiac rehabilitation (15 days in total). We derived the contents of patients’ experiences with modern technology from focus group interviews, the details of which have been presented in our previous study [43]. Half a year after the intervention, participants were divided into 4 categorized e-usage groups—*feeling outsider*, *being uninterested*, *reflecting benefit*, and *enthusiastic using*—which were based on the results of the qualitative data (Figure 1) [43].

### Participants

Qualitative and quantitative data were collected from participants at the rehabilitation center (10/39, 26% female; 29/39, 74% male). The participants' mean age was 54.8 (SD 9.4, range 34-77) years; 71% (27/38) participants had completed lower professional education. Of the 39 participants, 32 (82%) had undergone coronary angioplasty, and 4 (10%) had undergone coronary artery bypass surgery in the past 12 months before rehabilitation. Approximately 92% (25/27) of participants used the internet, and 37% (10/27) of participants used wrist activity trackers (Table 1 presents a description of participants at baseline by e-usage groups).

The e-usage groups of patients with cardiac disease—*feeling outsider*, *being uninterested*, *reflecting benefit*, and *enthusiastic using* [43]—were discovered in the same study population as in our previous study by using GT [43]. When we compared the groups’ background characteristics, only one statistically significant difference emerged. Mean age was signiﬁcantly diﬀerent among the groups (*P*=.003; analysis of variance [ANOVA] test). The *being uninterested* group participants were younger than participants in the other groups. The mean age of the *being uninterested* group was significantly lower than the mean age of the *feeling outsider* (mean difference [MD] −12.9; 95% CI −23.2 to −2.6; *P*=.009; Tukey honestly significant difference [HSD] test), *reflecting benefit* (MD −14.1; SD 4.15; 95% Cl −25.3 to −2.9; *P*=.009; Tukey HSD test) and *enthusiastic using* groups (MD −6.4; 95% Cl −12.6 to −0.2; *P*=.04; pairwise with 2-tailed *t* test).

**Table 1:** Description of participants at baseline by e-usage groups (N=39).

Description of participants			Feeling outsider (n=8)		Being uninterested (n=10)		Reflecting benefit (n=6)		Enthusiastic using (n=15)		Total
Age (years), mean (SD)			60.5 (7.6)		47.6 (5.6)		61.7 (11.2)		54 (8.2)		54.8 (9.4)
**Gender, n (%)**											
	Female	2 (25)		4 (40)		0		4 (27)		10 (26)	
	Male	6 (75)		6 (60)		6 (100)		11 (73)		29 (74)	
**Professional education, n (%)**											
	Lower education level	5 (63)		5 (56)		5 (83)		12 (80)		27 (71)	
	Higher education level	3 (38)		4 (44)		1 (17)		3 (20)		11 (29)	
**Time of heart operation, n (%)**											
	0-12 months before rehabilitation	5 (63)		6 (60)		4 (67)		10 (67)		25 (64)	
	Over 12 months before rehabilitation	2 (25)		2 (20)		1 (17)		5 (33)		10 (26)	
	No operations	1 (13)		2 (20)		1 (17)		0		4 (10)	
**Technology, n (%)**											
	Use internet	4 (80)		7 (100)		6 (100)		8 (89)		25 (93)	
	Use physical activity tracker	2 (40)		4 (57)		1 (17)		3 (33)		10 (37)	

### Intervention

The rehabilitation of patients with CHD occurred in three 5-day periods during the year. The aim of rehabilitation was to promote a patient’s adaptation to CHD and improve his or her functional capacity and ability to work [51]. A team of professionals included a physician, physical therapist, and nurse and optionally, a social worker, psychologist, or dietitian. For the remote component of the rehabilitation program, we used a secured remote coaching platform (m-coach Movendos) and an activity tracker accelerometer (Fitbit Charge HR). The 12-month web-based program involved feedback from each participant’s own physiotherapist. The program sent automatic motivational messages every month, and peer support was available in group discussions. Research participants set and monitored their health-related behavior goals by keeping a lifestyle and exercise diary and completing assignments.

### Data Collection

Data collection was guided by a purposeful sampling strategy called theoretical sampling in the GT method. This includes the purposeful selection of data samples to allow us to determine the variables that we would need to select to meet theoretical needs [44,45]. Table 2 presents the study’s biopsychosocial variable time points for collection.

Biomedical variables comprised measures such as waistline [52] and physical fitness (the 6-minute walk test [6MWT]) [53]. Physical activity was measured with a physical activity monitor of light-intensity physical activity using a Fitbit (Fitbit Inc) tracker [54] and the self-report International Physical Activity Questionnaire (IPAQ; 9 items) [55]. The World Health Organization Quality of Life-BREF (WHOQOL-BREF) questionnaire was used to assess individuals’ quality of physical health (domain 1). Other quality of life BREF domains are psychological health (domain 2), social relationships (domain 3), and the environment (domain 4) [56].

**Table 2:** Biopsychosocial variable time points for collection.

Biopsychosocial variables		Time point	
		0-month	12-month
**Biomedical**			
	Waistline [52]	✓	N/A^a^
	Physical fitness (6-minute walk test [6MWT]) [53]	✓	N/A
	Light-intensity physical activity accelerometer (LPA) [54]	✓	N/A
	International physical activity questionnaires (IPAQ) [55]	✓	N/A
	The World Health Organization Quality of Life-BREF (physical health, domain 1) [56]	✓	N/A
**Psychological**			
	Self-Efficacy to Regulate Exercise Scale (SERES) [57]	—^b^	✓
	The Behavioral Regulation in Exercise (BREQ-3) [58,59]	—	✓
	Questionnaire Depression Scale (DEPS) [60]	✓	N/A
	Quality of Life-BREF (psychological health, domain 2) [56]	✓	N/A
**Social**			
	Web-based participation (the number of task and message marks)	—^b^	✓
	Quality of Life-BREF (social relationships, domain 3, and environment, domain 4) [56]	✓	N/A

^a^N/A: not applicable.

^b^Data not available.

Psychological variables were measured using 3 questionnaires: quality of psychological health (WHOQOL-BREF, domain 2) [56,61], Self-Efficacy to Regulate Exercise Scale (SERES) based on SCT [57], and the Behavioral Regulation in Exercise Questionnaire (BREQ-3). BREQ-3 is a 24-question instrument and is based on self-determination theory [58,59]. The Depression Scale (a 10-item DEPS) [60] was also included to measure psychological variables.

Social variables comprised participation in the web-based program and the quality of life questionnaire. Participation in the program was measured by individuals’ visits to the site, including the number of pages they visited, the number of tasks they had completed (the number of completed task marks), and the number of conversations they had participated in (the number of message marks) during the 12 months of intervention. Social preintervention variables were also included in the questionnaire responses regarding the quality of social relationships and the environment (WHOQOL-BREF; domain 3 and domain 4) [56].

### Data Analysis

The constant comparative method of the classic GT [44,45] guided the data analysis. That is, we analyzed data for similarities and differences at a more abstract level to move toward substantive theory building [44,46,47]. We recorded our research group’s reflective discussions and wrote both theoretical and analytical memos. Memos were seen as a link between the research group’s notions and theoretical ideas, and they helped us in data analysis and meaning interpretation. In the following paragraphs, we describe our quantitative analysis and use of a mixed methods GT approach.

**Figure 2:**
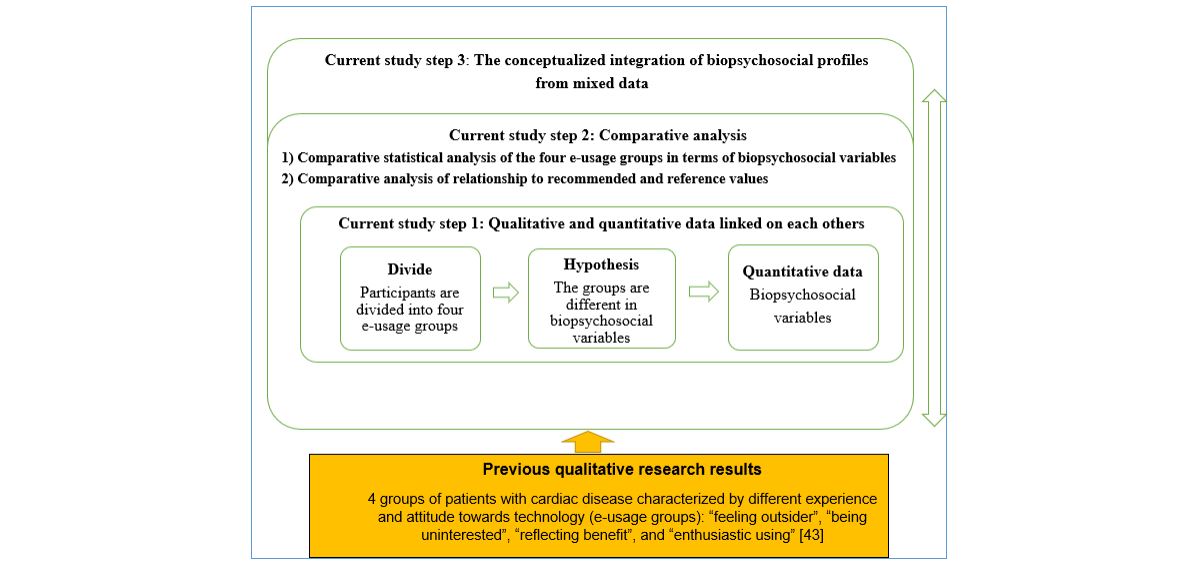
Three-step analyses process.

In our previous study, we analyzed interview data using GT. The result of the qualitative study was 4 e-usage groups—*feeling outsider*, *being uninterested*, *reflecting benefit*, and *enthusiastic using* [43]. In the first step, we divided participants into these 4 e-usage groups. A total of 2 researchers (MRA, HK) in our study independently read the interview responses of the participants. These researchers independently divided participants into 4 e-usage groups, taking into account the qualitative descriptions of the different e-usage groups: (1) technology experience, (2) attitude, and (3) expectations of remote counseling. There was moderate agreement between the 2 researchers in the coding of responses into the groups, κ=0.521 [62]. The 2 researchers compared their divided results, discussed disagreements, and reanalyzed the disagreed-upon results together. The results were also discussed with a third researcher (TS) to finalize the coding results. On the basis of our previous qualitative results [43], we presented a hypothesis, selected available biopsychosocial variables, and used quantitative methods and techniques to promote the generation of a substantive theory [43,44]. Figure 2 describes the entire three-step analysis.

In the second step, statistical analyses were used to examine the differences in biopsychosocial variables among the 4 groups. All quantitative data analyses were performed using the SPSS (version 24, SPSS Inc). We report descriptive statistics for the variables being compared. We examined the differences in biopsychosocial variables among the groups with probability statistics (*P*<.05) to determine whether the proposed differences within the group could be confirmed. As a measure of precision for the estimate, a 95% CI was reported.

ANOVA, *t* test, or nonparametric test (Mann–Whitney and Kruskal–Wallis tests) was used when appropriate. Thereafter, pairwise comparisons between the groups were analyzed with ANOVA (the Tukey HSD test, the Kruskal–Wallis or Mann–Whitney test (with Bonferroni correction). For comparisons of three or more group means, we performed the (one-way) ANOVA or nonparametric Kruskal–Wallis test. ANOVA was only used if the data in each group were normally distributed and the variances were homogeneous. Normality of the groups was assessed by the Shapiro–Wilk test, as all group sizes were <50. Homogeneity of variances was evaluated by the Levene test. When we had a significant result for differences between group means in the main test, we performed post hoc comparisons. For ANOVA, we applied the Tukey test, and for the Kruskal–Wallis test, we used the Mann–Whitney pairwise comparisons while adjusting the significance values by the Bonferroni correction for multiple tests.

After completing the series of quantitative analyses, we compared the results of the 4 groups in terms of recommended and reference values. We compared physical activity level (accelerometer and questionnaire) with World Health Organization’s global recommendations for physical activity for health, that is, 150 minutes each week [54] and the quality of life questionnaire results with averages for the Finnish population (aged 18-98 years) [61]. In the DEPS (0-30), the cutoff point for depression is ≥8 points, which indicates sensitivity to depression [60]. The questionnaire (SERES and BREQ-3) results were compared with the mean value of the scale. The mean value of SERES is 50 (0-100) [57] and that of BREQ-3 is 0 (−24 to 24) [58,59]. We compared the number of completed remote tasks and messaging markings with the total sample mean values of participation in the web-based program (the number of completed tasks was 87 for remote tasks and 6.6 for messaging).

In the final step of the analysis, a constant comparison was performed conceptually by analyzing the meanings behind the numbers for discovering and generating substantive theory based on GT [44,45]. Quantitative data were compared systematically by theoretical coding variables within groups. We grounded profile conceptualization by critically examining and questioning the data, which was theoretically sensitive. Finally, we formed biopsychosocial profiles based on the integrated findings of the constant comparative analysis phases. On the basis of these conceptualization processes, we renamed the profile of each group and formed the main category (Table 3 shows an example of a constant comparative analysis process in the *feeling outsider* group).

The results of this study’s quantitative phase align with our qualitative findings. Our analyses moved toward substantive theory when we performed a constant comparative analysis of the qualitative and quantitative data [45,46]. As Glaser stated, “it is important to fully understand the meaning behind the numbers and techniques when using quantitative data [45].” The following paragraphs describe the results of the intermediate stages of comparative analyses in more detail.

**Table 3:** The feeling outsider group constant comparative analysis (n=8)^a^.

Variables	Feeling outsider, mean (SD)	Result in significant differences between groups	RV^b^	Values, mean/RV (%)	Profile descriptions^c^
Waistline (centimeters)	107.1 (11.1)	—^d^	<94	+13.9	The *feeling outsider* group had high-risk behavior related to overweight
6-minute walk test (meters)	575.5 (73.3)	The *feeling outsider* group had lower physical fitness (*P*=.047) than the *enthusiastic using* group.	>623	–7.6	The *feeling outsider* group had high-risk behavior related to being inactive
Light-intensity physical activity, accelerometer (n=6)	134.9 (58.6)	—	>150	–10	Self-reported weekly physical activity differed from accelerometer-measured physical activity
IPAQ^e^ (n=7)	421.4 (468.4)	—	>150	+280.9	Self-reported weekly physical activity differed from accelerometer-measured physical activity
WHOQOL-BREF^f^ physical health	13.6 (2.9)	—	>16.5	–17.6	Physical quality of life was low at the beginning of rehabilitation
Self-Efficacy to Regulate Exercise Scale (0-100; n=7)	67.0 (19.2)	—	>50	+34	They had a high self-efficacy to regulate exercise at the end of the rehabilitation according to their own estimate
The number of completed task mark^g^	45 (126.1)	Performing guided tasks in the program (*P*=.03) were lower in the *feeling outsider* group than in the *enthusiastic using* group	>87	–48.3	Their engagement in technological solution was low
The number of discussions mark^g^	4.3 (7.6)	Communicating via messages (*P*=.03) were lower in the *feeling outsider* group than in the *enthusiastic using* group	>6.6	34.8	Their engagement in technological solution was low

^a^Hypothesis: There would be differences between the 4 e-usage groups *feeling outsider*, *being uninterested*, *reflecting benefit*, and *enthusiastic using* [43]. Proposition: The *feeling outsider* group might benefit from developing self-efficacy in physical activity and adequate positive support, as individuals in this group consider themselves as outsiders and find technology fearsome.

^b^RV: recommended value.

^c^On the basis of these results, a profile for the group *feeling outsider* was renamed *building self-awareness*.

^d^No significant differences between the *feeling outsider* and others e-usage groups.

^e^IPAQ: International Physical Activity Questionnaire.

^f^WHOQOL-BREF: The World Health Organization Quality of Life Questionnaire, Short Form.

^g^Postintervention variables.

## Results

### Comparative Statistical Analysis of the 4 e-Usage Groups in Terms of Biopsychosocial Variables

The results of the comparative analysis provide an understanding of the biopsychosocial profiles of e-usage groups.

Statistically significant differences (*P*<.05) between groups were found for the biomedical variable waistline, which significantly differed between the *being uninterested* and *enthusiastic using* groups (MD 14.2; 95% CI 1.0 to 27.5; *P*=.03; Tukey HSD test). The *being uninterested* group had a larger waistline than the *enthusiastic using* group. The 6MWT also showed signiﬁcant differences between *being uninterested* and *enthusiastic using* groups (MD −0.72; 95% CI −1.4 to −0.06; *P*=.03; Tukey HSD test) and between the *feeling outsider* and *enthusiastic using* groups (MD 55.8; 95% CI −110.7 to −0.92; *P*=.047; 2-tailed *t* test). The *feeling outsider* group had lower physical fitness than the *enthusiastic using* group. For the biomedical variables, light-intensity physical activity and IPAQ, there were no significant differences among the 4 groups, and the psychological and social variables, DEPS and quality of social life, were also nonsignificant (Table 4).

The results for the postintervention variables are presented next. The BREQ-3 scores were signiﬁcantly diﬀerent between the *being uninterested* and *enthusiastic using* groups in a *t* test (MD −7.3; 95% CI −13.5 to −1.1; *P*=.02); the degree of self-determination in exercise was lower for the former than for the latter. The results for SERES were nonsignificant. Participation in the web-based program (0-12 months) was the only statistically significant difference in group comparisons, with task marking diﬀering significantly. Pairwise comparison revealed signiﬁcant diﬀerences. Performing guided tasks in the program in the Kruskal–Wallis test (*P*=.04) and communicating via messages were lower in the *feeling outsider* group than in the *enthusiastic using* group (*P*=.03) in the Mann–Whitney test (Table 5).

**Table 4:** Comparative quantitative analysis among the 4 groups in terms of biopsychosocial preintervention variables.

Biopsychosocial variables preintervention		Group 1, *feeling outsider* (n=8)			Group 2, *being uninterested* (n=10)			Group 3, *reflecting Benefit* (n=6)			Group 4, *enthusiastic using* (n=15)	
		Value, mean (SD)	n (%)	Value, mean (SD)		n (%)	Value, mean (SD)		n (%)	Value, mean (SD)		n (%)
**Biomedical variables**												
	Waistline (centimeters) [52]	107.1 (11.1)	—^a^	112.7^b^ (13.6)		—	102.3 (12.3)		—	98.4^b^ (11.3)		—
	6-minute walk test (meters) [53]	575.5^b^ (73.3)	—	558.9^c^ (61.1)		9 (90)	624.3 (28.8)		4 (67)	631.3^b,c^ (52.7)		—
	Light-intensity physical activity, accelerometer (minutes/week) [54]	134.9 (58.6)	6 (75)	174.6 (48.8)		6 (60)	137.2 (49.1)		4 (67)	148.3 (59.8)		13 (87)
	The International Physical Activity Questionnaire (min/week) [55]	421.4 (468.4)	7 (88)	461.3 (445.5)		8 (80)	320.8 (411.4)		—	291.0 (307.4)		—
	WHOQOL-BREF^d^ physical health (4-20) [56]	13.6 (2.9)	—	13.7 (2.2)		—	14.4 (2.3)		—	14.2 (2.2)		—
**Psychological variables**												
	WHOQOL-BREF psychological health (4-20) [56]	14.3 (2.7)	—	14.2 (2.2)		—	14.4 (3.0)		—	15.5 (1.9)		—
	The Depression Scale (0-30) [60]	6.8 (5.9)	6 (75)	6.7 (5.3)		—	2.0 (1.9)		5 (83)	4.2 (3.9)		—
**Social variables**												
	WHOQOL-BREF social relationship (4-20) [56]	14.3 (2.5)	—	15.9 (2.5)		—	15.7 (1.9)		—	16.3 (2.9)		—
	WHOQOL-BREF environment (4-20) [56]	14.9 (2.6)	—	14.3 (2.3)		—	15.3 (1.7)		—	15.0 (2.2)		—

^a^No missing data.

^b^Significant difference (*P*<.05) among groups.

^c^Significant difference (*P*<.05) among groups.

^d^WHOQOL-BREF: The World Health Organization Quality of Life Questionnaire, Short Form.

**Table 5:** Comparative quantitative analysis among the 4 groups in terms of biopsychosocial postintervention variables.

Biopsychosocial variables postintervention		Group 1, *feeling outsider* (n=8)			Group 2, *being uninterested* (n=10)			Group 3, *reflecting benefit* (n=6)			Group 4, *enthusiastic using* (n=15)	
		Value, mean (SD)	n (%)	Value, mean (SD)		n (%)	Value, mean (SD)		n (%)	Value, mean (SD)		n (%)
**Biomedical variables**												
	The number of completed tasks mark	45^a^ (126.1)	—^b^	31.4 (48.4)		—	116.8 (142.8)		—	156.0^a^ (204.7)		—
	The number of discussions mark	4.3^a^ (7.6)	—	6.1 (4.2)		—	7.8 (8.0)		—	8.1^a^ (6.9)		—
	Self-Efficacy to Regulate Exercise Scale (0-100) [57]	67.0 (19.2)	7 (88)	56.6 (18.3)		7 (70)	62.0 (9.2)		—	54.2 (17.4)		14 (93)
	The Behavioral Regulation in Exercise Questionnaire 3 (−24 to 24) [58,59]	12.0 (8.3)	6 (75)	5.7^a^ (8.0)		7 (70)	11.8 (2.1)		—	13.1^a^ (5.5)		14 (93)

^a^Indicates significant difference (*P*<.05) among the groups.

^b^No missing data.

### Comparative Analysis of Relationship to Recommend and Reference Values

We compared the results of the 4 groups in terms of recommended and reference values. All e-usage groups had larger waistline and lower 6MWT values compared with the risk of disease cutoff values (waistline <94/6MWT >623); *feeling outsider* (mean 107.1/mean 575.5), *being uninterested* (mean 112.7/mean 558.9), *reflecting benefit* (mean 102.3/mean 624.3), and *enthusiastic using* (mean 98.4/mean 631.3). Regarding the quality of social relationships (>16.5), the *feeling outsider* (mean 14.3) and *being uninterested* (mean 15.9) groups reported lower quality of social relationships than that of the *reflecting benefit* (mean 15.7) and *enthusiastic using* (mean 16.3) groups. Except for the *enthusiastic using* group, which had near-average values, the quality of life results for all groups were lower than the average values for the Finnish population (Table 6).

The self-efficacy values of all groups were better than the mean value of the scale (>50). On the other hand, the opposite results were observed for variables of exercise self-efficacy, in which the *enthusiastic using* group had lower self-efficacy (mean 54.2) than the *feeling outsider* (mean 67), *being uninterested* (mean 56.6) and *reflecting benefit* (mean 62; Table 7) groups.

**Table 6:** Comparative analysis of relationship to recommended and reference values (preintervention).

Biopsychosocial preintervention variables		RV^a^	*Feeling outsider* (n=8)		*Being uninterested*, mean (n=10)			*Reflecting benefit* (n=6)			*Enthusiastic using* (n=15)	
			Values, mean (SD)	Level of factor, mean/RV (%)	Values, mean (SD)	Level of factor, mean/RV (%)	Values, mean (SD)		Level of factor, mean/RV (%)	Values, mean (SD)		Level of factor, mean/RV (%)
**Biomedical variables**												
	Waistline (centimeter) [52]	<94	107.1 (11.1)	+13.9	112.7 (13.6)	+19.9	102.3 (12.3)		+8.8	98.4 (11.3)		+4.7
	Physical fitness (meter) [53] (6-minute walk test)	>623	575.5 (73.3)	−7.6	558.9 (61.1)	−10.3	624.3 (28.8)		+0.2	631.3 (52.7)		+1.3
	Light Physical activity, accelerometer (minutes/week) [54]	>150	134.9 (58.6)	−10	174.6 (48.8)	+16.4	137.2 (49.1)		−8.5	148.3 (59.8)		−1.1
	The International Physical Activity Questionnaires (minutes/week) [55]	>150	421.4 (468.4)	+280.9	461.3 (445.5)	+307.5	320.8 (411.4)		+213.9	291 (307.4)		94
	WHOQOL-BREF^b^ Physical health [56]	>16.5	13.6 (2.9)	−17.6	13.7 (2.2)	−17.0	14.4 (2.3)		−12.7	14.2 (2.2)		−13.9
**Psychological variables**												
	WHOQOL-BREF psychological health [56]	>15.5	14.3 (2.7)	−7.7	14.2 (2.2)	−8.4	14.4 (3.0)		−7.1	15.5 (1.9)		0
	The Depression Scale (0-30) [60]	<8	6.8 (5.9)	−15	6.7 (5.3)	−16.3	2.0 (1.9)		−75	4.2 (3.9)		−47.5
**Social variables**												
	WHOQOL-BREF social relationship [56]	>16.5	14.3 (2.5)	−13.3	15.9 (2.5)	−3.6	15.7 (1.9)		−4.8	16.3 (2.9)		−1.2
	WHOQOL-BREF environment [56]	>16.5	14.9 (2.6)	−9.7	14.3 (2.3)	−13.3	15.3 (1.7)		−7.3	15.0 (2.2)		−9.09

^a^RV: recommended value.

^b^WHOQOL-BREF: The World Health Organization Quality of Life Questionnaire, Short Form.

**Table 7:** Comparative analysis of relationship to recommended and reference values (postintervention).

Biopsychosocial postintervention variables			RV^a^		*Feeling outsider* (n=8)					*Being uninterested* ( (n=10)					*Reflecting benefit* (n=6)					*Enthusiastic using* (n=15)		
					Values, mean (SD)		Level of factor, mean/RV (%)		Values, mean (SD)			Level of factor, mean/RV (%)		Values, mean (SD)			Level of factor, mean/RV (%)		Values, mean (SD)			Level of factor, mean/RV (%)
**Postintervention variables**																						
	Task marks	>87		45 (126.1)		−48.3		31.4 (48.4)			−63.9		116.8 (142.8)			+34.3		156.0 (204.7)			+79.3	
	Discussion marks	>6.6		4.3 (7.6)		−34.8		6.1 (4.2)			−7.6		7.8 (8.0)			+18.2		8.1 (6.9)			+22.7	
	Self-Efficacy to Regulate Exercise Scale [57]	>50		67.0 (19.2)		+34		56.6 (18.3)			+13.2		62.0 (9.2)			+24		54.2 (17.4)			+8.4	
	The Behavioral Regulation in Exercise Questionnaire [58,59]	>0		12.0 (8.3)		+12		5.7 (8.0)			+5.7		11.8 (2.1)			+11.8		13.1 (5.5)			+13.1	

^a^RV: recommended value.

### Conceptualized Integration of Biopsychosocial Profiles From Mixed Data

The results were synthesized to build the biopsychosocial profiles for the 4 groups—*feeling outsider*, *being uninterested*, *reflecting benefit*, and *enthusiastic using*—as part of the rehabilitation process. We formed biopsychosocial profiles based on constant comparative analysis through narrative description.

Proposition 1: The *feeling outsider* group might benefit from developing self-efficacy in physical activity and adequate positive support, as individuals in this group consider themselves as outsiders and find technology fearsome:

That technology hasn’t really come [...] My wife taught the computer [...] supported, well, taught—so I went to the courses. And the kids did. I thought that if I’m still starting to tinker, there won’t be enough hours in the day to learn [43].

participant 25, 60-year-old man, focus group 1

The *feeling outsider* group had high-risk behavior related to being inactive and overweight. Self-reported weekly physical activity differed from accelerometer-measured physical activity. In addition, physical quality of life was low at the beginning of rehabilitation. Members of this group had a high self-efficacy to regulate exercise at the end of the rehabilitation according to their own estimate; however, their engagement in technological solutions was low. Their biomedical results were inconsistent between their self-reported physical activity and objectively measured data, which may have been because of a lack of lifestyle self-awareness. On the basis of these results, the profile for the *feeling outsider* group was renamed *building self-awareness*

Proposition 2: The b*eing uninterested* group might benefit from weight management and physical activity self-monitoring with reminders and prompts, as they feel externally motivated:

I’m waiting for it and I’m truly interested, as if I were waiting for something like a spark. That it is something, something like, motivating, and...well...I can’t say, but it like maybe not now for sure every week. If once a month, certainly something could come...a reminder [43].

participant 56, 45-year-old man, focus group 3

When I could enter inputs in there, and if my own activities could be there, then I would be like a response: Is this the right or wrong direction, and...And that’s when it’s really somebody, something and someone monitoring what you’re doing [43].

participant 41, 49-year-old woman, focus group 2

The *being uninterested* group had low levels of physical fitness, poor self-assessed physiological quality of life, and a high waist circumference. Their exercise behavior can be described as externally regulated, with low scores in self-determination. In addition, they were interested in self-monitoring their physical activity but were uninterested in participating in web-based coaching. Their self-monitoring technology may have motivated them to improve their physical activity levels and engagement in lifestyle changes. The profile for the *being uninterested* group was renamed *increasing engagement.*

Proposition 3: The *reflective benefit* groups might benefit from easy-to-use and interactive technology, as their interest is maintained by technology with personalized information and interactive tracking tools:

Let’s put it in this way: I’m not actually now that way from being pushed, yeah. Yes it comes from my own desire. The main purpose is monitoring: it’s for that. It’s interesting to follow what happens if you change some exercise habits, and you can see from this, what changes have happened in the background. Very okay [43].

participant 17, 57-year-old man, focus group 2

The *reflecting benefit* group showed healthy lifestyle choices related to eating behavior and exercise. They may have had intrinsic motivation for exercise and high self-determination, including a positive balance in life. Higher scores indicated higher self-efficacy for exercise and health technology interest. Their biopsychosocial outcomes were balanced and maintaining these outcomes could be the most important goal for them. The *reflecting benefit* group was renamed *maintaining a healthy lifestyle balance*.

Proposition 4: The *enthusiastic users* group might benefit from empowering their self-efficacy and personalized lifestyle feedback, as they have a positive technology mastery experience:

I’m waiting and I’m interested. Yes, of course, this here now gives little push in the pants. I’m already moving pretty well, that’s what this thing around my arm tells me...Yeah...and then yes, I have the Sport Tracker on my phone, also. When I go somewhere, I tell it to draw a map, and I see the time and all that [43].”

participant 66, 34-year-old man, focus group 3

Modern opportunities. And if now, of course...from where soon could come a little spark, and that spark continues than exercise could begin. And it’s really the same benefit. And then, of course, if nothing’s heard from there. It sounds real good, and then reminders. Something like you can write comments, and [...] [43]

participant 26, 61-year-old woman, focus group 2

**Figure 3:**
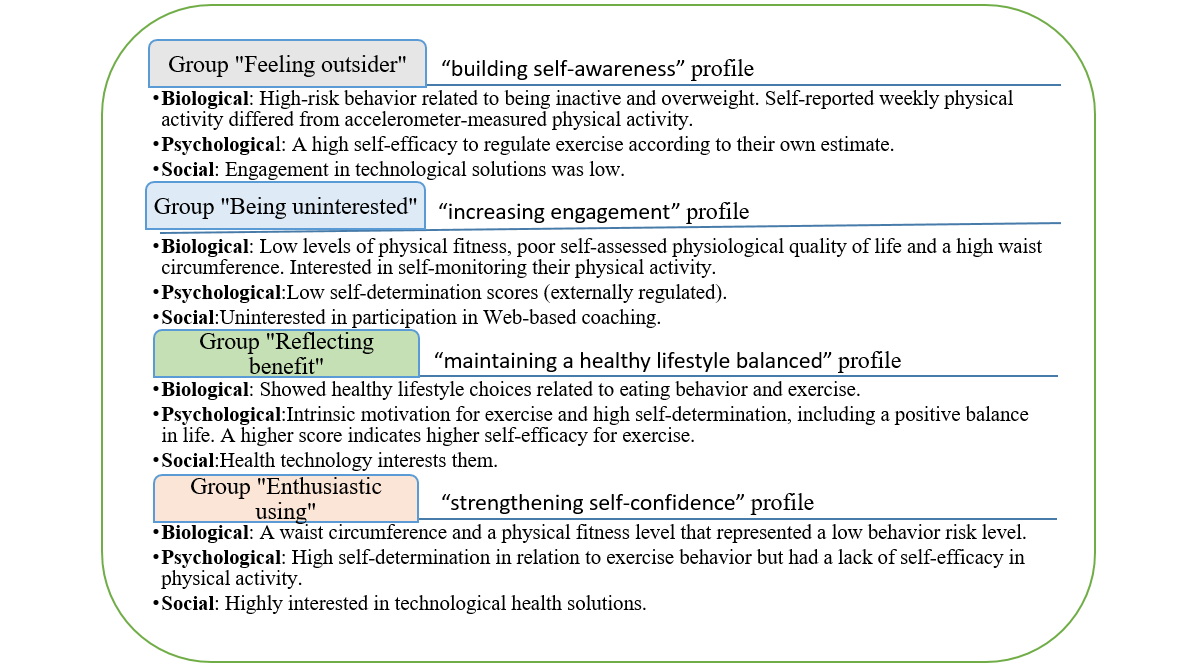
Group biopsychosocial profile descriptions.

The *enthusiastic using* group had a waist circumference and a physical fitness level that represented a low behavior risk level. They had high self-determination in relation to exercise behavior but lacked self-efficacy in physical activity. They were highly interested in technological health solutions. This group had a healthy lifestyle; however, their physical self-efficacy related to exercise was low. A heart event may have lowered their self-confidence in health behaviors. The profile for the *enthusiastic using* group was renamed *strengthening self-confidence.* Figure 3 shows a summary of the groups’ similarities and differences in the comparative analysis results.

**Figure 4:**
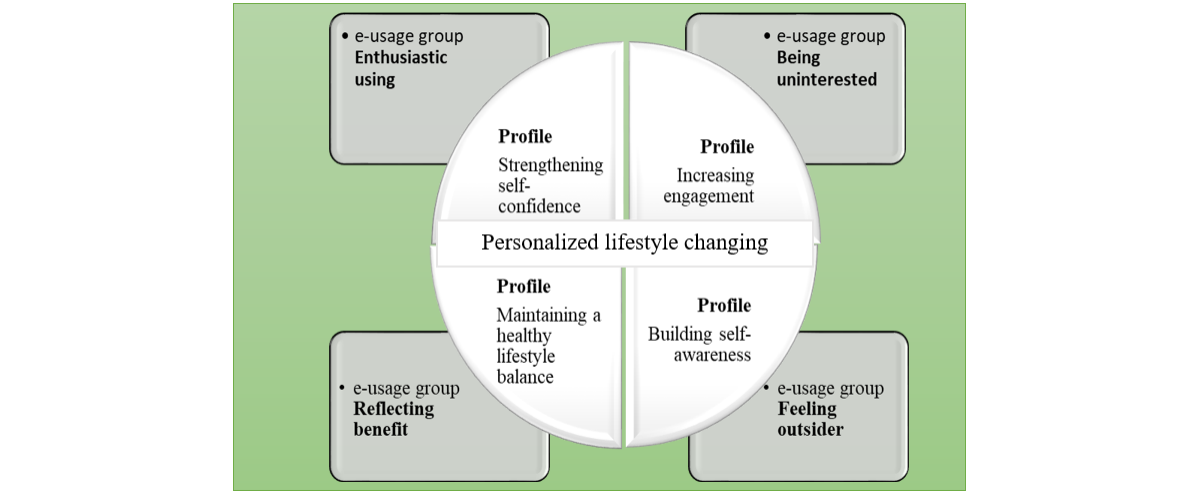
Biopsychosocial personalized lifestyle changing profiles in relation to the rehabilitation process.

On the basis of these results, we were able to synthesize all groups’ biopsychosocial profile descriptions to a thematic meaning, that is, *personalized lifestyle changing as part of the rehabilitation process*, which can be the start of substantive theory development integrated into all 4 groups’ profile descriptions. On the basis of the analysis, we identified and renamed the 4 groups to *building self-awareness*, *increasing engagement*, *maintaining a healthy lifestyle balance,* and *strengthening self-confidence*. These profiles showed how individuals in the 4 groups identified their different lifestyle management reflections in rehabilitation progress. The main results of the analysis are shown in Figure 4.

## Discussion

### Principal Findings

The main result of the study was *personalized lifestyle changing as part of the rehabilitation process,* which refers to the 4 groups’ profiles related to rehabilitation progress. On the basis of the qualitative and quantitative GT analysis, we identified 4 profiles: *building self-awareness*, *increasing engagement*, *maintaining a healthy lifestyle balance*, and *strengthening self-confidence*. The main message of this study is that it is important to identify different biopsychosocial profiles with respect to the reflections of patients with cardiac disease on their lifestyle risk factor management in the counseling process of remote rehabilitation. This knowledge can give cardiac rehabilitation professionals evidence and enable them to tailor theory-based web-based behavior change interventions.

Patients in the *feeling outsider* group were afraid to use technology, and they expected supportive behavior change counseling [43]. This group, with the *building self-awareness* profile, had low daily physical activity and was overweight. In their self-reports, members of this group overestimated the amount of physical activity relative to their objectively measured data. A possible explanation for these results may be their lack of self-awareness concerning self-management of lifestyle risk factors. However, studies have shown a higher estimate of physical activity using the IPAQ than the accelerometer data [63]. Self‐management skills and attitudes included in lifestyle change are based on motivational, goal-setting, controlling, and self-regulatory skills, which require self-awareness [64]. Although promoting the ability to recognize how self-efficacy, thoughts, feelings, and actions are interconnected, rehabilitation also improves self-awareness for self-management of lifestyle change processes [30,31,33,64,65]. The group with this profile needs guidance and positive support in using technology [43] and in increasing their self-awareness. Patients in this group may benefit from web-based goal-setting tools for self-awareness. Goal setting could help these patients identify their own risk factors and set realistic and meaningful goals. Health professionals should take into account patients’ aims, needs, and self-efficacy, as well as health outcome information in individual goal-setting.

The *being uninterested* group expected problem-free technology with activity-empowering web-based counseling [43]. This group, with the *increasing engagement* profile, had lower self-efficacy, and they might have quickly given up when they ran into difficulties [43]. In addition, we found that the group was uninterested in participating in web-based coaching. However, members showed interest in tracking their physical activity with a wearable accelerometer. Patients in this group showed low scores in self-determination, and thus, their motivation can be described as externally regulated. Previous studies have reported that regular physical activity can reduce cardiovascular risk factors [1-4]. Activity tracking accelerometers with feedback may boost self-efficacy, which has been shown to promote cardiovascular risk self-management [29-31]. Wearing an accelerometer itself may promote and motivate physical activity [66]. Patients in this group had low levels of physical fitness, poor self-assessed physiological quality of life, and high waist circumferences. Previous research has shown that biopsychosocial characteristics are related to lower scores in risk factor self-management, especially in women [6,37,41]. Additional support can be provided using evidence-based health behavior change techniques with the help of technology in rehabilitation [15,30,32,65]. Patients in this group may benefit from support and guidance to increase their engagement in lifestyle-changing processes. Health professionals should take into account such patients’ motivations to use self-monitoring technology and their interests in personalized and regular feedback, reminders, and prompts.

Patients in the *reflecting the benefit* group expected easy-to-use and useful technology with interactive tools [43]. The group showed healthy lifestyle choices, such as healthy eating and exercising. These patients had high self-efficacy in achieving physical activity goals, and they were interested in health technology. This group, with a *maintaining a healthy lifestyle balance* profile, had a fair amount of intrinsic motivation for exercise and high self-determination for exercise behavior, which is needed to increase self‐management skills and facilitate lifestyle change [64,67]. Self-monitoring and realistic goal setting are important factors in the process of self-regulation [10,16]. Our findings indicate that increases in regular exercise competence could improve intrinsic motivation, as shown in previous studies [35,36]. Patients with this profile may benefit from interactive and easy-to-use tracking tools through which self-monitoring allows them to manage their health. Health professionals should monitor the goal progress to meet their desired functional goals.

The *enthusiastic using* group expected smoothly functioning technology that offered empowering self-tracking with feedback [43]. This group had minor risk behavior but the lowest self-efficacy in physical activity compared with the other group profiles. The results of Kärner Köhler et al [68] indicate that self-efficacy is not related to chronic conditions. However, a cardiac event may have reduced these patients’ self-confidence in their own lifestyle management. They may not have believed in their own behavior choices for reaching the desired goal. A possible explanation might be that patients conscientiously followed a healthy lifestyle. A previous study showed that people with higher conscientiousness were more intrinsically motivated [35]. Early self-efficacy support may improve individuals’ participation in web-based programs [31,33]. Patients with the *strengthening self-confidence* profile may benefit from early self-management support for self-confidence. Health professionals should provide support, especially in the early stages after heart events, by focusing on positive achievements.

The profiles showed how patients in the 4 groups adjusted their lifestyles differently on the part of rehabilitation progress. Patients in the *feeling outsider* and *being uninterested* groups had high-risk behavior and low engagement in technological solutions. In contrast, patients in the *reflecting benefit* and *enthusiastic using* group profiles had low-risk behavior and good adherence to web-based interventions. Biopsychosocial profiles have been used to tailor interventions for patients with chronic pain [38,39], diabetes [40], overweight and obesity [41], and hypertension [42]. It is also important to identify the biopsychosocial profiles of patients with cardiac disease, as it allows for evidence- and theory-based and individually tailored lifestyle counseling programs in multidisciplinary fields.

### Limitations and Strengths

This study has some limitations related to the sample size, which was unevenly distributed among the groups. The purpose of the study was theoretical verification using GT, and for this purpose, there was an inductive generalization regarding the phenomenon under study and no statistical generalization. We have provided detailed descriptions that were not intended for extrapolation of the findings to other settings but to provide information about the phenomenon and build substantive theory. The possible sampling bias, small sample size, and sampling strategy certainly limited our quantitative analyses; however, we used GT and mixing methods of constant comparative analysis, which was beneficial to our study when we grounded several variables. This study was based on GT, and the results can be said to be reliable based on thick descriptions, taking into account thorough descriptive information about the study setting, study participants, and processes. There are weaknesses in this study; for example, we collected data from the BREQ-3 and SERES questionnaires only at the end of the intervention. It would have been better if all questionnaire data had also been collected preintervention. However, despite this shortcoming, the BREQ-3 and SERES questionnaires provided valuable information. The mixing of methods was an innovative challenge. The credibility of the results was based on conceptualization to enable a greater understanding of patient experiences with technology in the context of digital cardiac rehabilitation. There is also a need for information on whether there might be a change in patients' experiences and attitudes toward technology during rehabilitation. The implementation of these results might be useful, especially in the planning of rehabilitation counseling and teaching.

### Conclusions

The study showed that *personalized lifestyle changing as part of the rehabilitation process* relates to the profile descriptions of the 4 groups. On the basis of the profiles, we identified 4 profiles related to the rehabilitation process: *building self-awareness*, *increasing engagement*, *maintaining a healthy lifestyle balance*, and *strengthening self-confidence*. The results might help to understand what is meaningful for Finnish patients with cardiovascular disease who participate in a rehabilitation program with face-to-face and remote web components. The personalized behavior change components can be embedded in the technology part of cardiac rehabilitation, for example, individual goal setting, self-monitoring, reminders and prompts, positive social and peer group support, personalized information, and feedback. These components increase the *spark* for motivation to a lifestyle change by taking into account the different life situations, needs, and concerns of individuals and their experiences and attitudes toward the use of technology. However, future studies are needed that back up our current results with larger sample sizes and a sociodemographic structure that mirrors the study population.

## Abbreviations

6MWT: 6-minute walk test
ANOVA: analysis of variance
BREQ-3: The Behavioral Regulation in Exercise-3
CHD: coronary heart disease
DEPS: the Depression Scale
GT: grounded theory
HSD: honestly significant difference
IPAQ: The International Physical Activity Questionnaire
MD: mean difference
SCT: social cognitive theory
SERES: Self-Efficacy to Regulate Exercise Scale
WHOQOL-BREF: The World Health Organization Quality of Life-BREF
